# Migration Dynamics of Fall Armyworm *Spodoptera frugiperda* (Smith) in the Yangtze River Delta

**DOI:** 10.3390/insects14020127

**Published:** 2023-01-26

**Authors:** Xue-Yan Zhang, Le Huang, Jie Liu, Hai-Bo Zhang, Kun Qiu, Fang Lu, Gao Hu

**Affiliations:** 1 College of Plant Protection, Nanjing Agricultural University, Nanjing 210095, China; 2 China National Agro-Tech Extension and Service Center, Beijing 100125, China; 3 Plant Protection and Plant Quarantine Station of Jiangsu Province, Nanjing 210036, China; 4 Plant Protection Station of Anhui Province, Hefei 230031, China; 5 Shanghai Agricultural Technology Extension and Service Center, Shanghai 201103, China

**Keywords:** Yangtze River Delta, fall armyworm, migration, trajectory analysis

## Abstract

**Simple Summary:**

Fall Armyworm *Spodoptera frugiperda* (Smith) is one of the most important agricultural pests, which seriously threatens agricultural production and food security in China. The Yangtze River Delta is an important passage for the northward migration of *S. frugiperda* in eastern China, connecting the year-round breeding area in South China and the major maize planting area in Northern China. Understanding the migration dynamics in this area is crucial for the forecasting and controlling of this pest throughout the country. Based on three years’ data (2019–2021) in the Yangtze Delta, the migration timing, migration pathway, the source area of immigrants and the potential landing area of emigrants were identified. It revealed that *S. frugiperda* mainly migrated into the Yangtze River Delta in May and June, and their progenies further migrated northward to Northern China mostly in July and August. The results of this paper will be helpful to further understand the migratory rules of *S. frugiperda*, and to provide a theoretical basis for pest forecasting and pest control.

**Abstract:**

The Yangtze River Delta, located in East China, is an important passage on the eastern pathway of the northward migration of fall armyworm *Spodoptera frugiperda* (Smith) in China, connecting China’s year-round breeding area and the Huang-Huai-Hai summer maize area. Clarifying the migration dynamics of *S. frugiperda* in the Yangtze River Delta is of great significance for the scientific control and prevention of *S. frugiperda* in the Yangtze River Delta, even in the Huang-Huai-Hai region and Northeast China. This study is based on the pest investigation data of *S. frugiperda* in the Yangtze River Delta from 2019 to 2021, combining it with the migration trajectory simulation approach and the synoptic weather analysis. The result showed that *S. frugiperda* migrated to the Yangtze River Delta in March or April at the earliest, and mainly migrated to the south of the Yangtze River in May, which can be migrated from Guangdong, Guangxi, Fujian, Jiangxi, Hunan and other places. In May and June, *S. frugiperda* migrated further into the Jiang–Huai region, and its source areas were mainly distributed in Jiangxi, Hunan, Zhejiang, Jiangsu, Anhui and Hubei provinces. In July, it mainly migrated to the north of Huai River, and the source areas of the insects were mainly distributed in Jiangsu, Anhui, Hunan, Hubei and Henan. From the south of the Yangtze River to the north of the Huai River, the source areas of *S. frugiperda* were constantly moving north. After breeding locally, *S. frugiperda* can not only migrate to other regions of the Yangtze River Delta, but also to its surrounding provinces of Jiangxi, Hunan, Hubei, Henan, Shandong and Hebei, and even cross the Shandong Peninsula into Northeast China such as Liaoning and Jilin provinces. Trajectory simulation showed that the emigrants of *S. frugiperda* from the Yangtze River Delta moved northward, westward and eastward as wind direction was quite diverse in June–August. This paper analyzes the migration dynamics of *S. frugiperda* in the Yangtze River Delta, which has important guiding significance for the monitoring, early warning and the development of scientific prevention and control strategies for whole country.

## 1. Introduction

Fall armyworm, *Spodoptera frugiperda* (Smith), originates from tropical and subtropical areas of America [1,2,3]. It was first found outside its origin in Nigeria, Africa, in January 2016, and has since spread rapidly across African countries [3,4,5]. In 2018, *S. frugiperda* invaded Asia and was successively found in India, Myanmar, Laos, Vietnam and other neighboring countries [6]. Then, *S. frugiperda* invaded Jiangcheng County, Yunnan Province, China in November 2018 [7,8]. In the following years, it was found in more than 1500 counties of 27 provinces (municipalities and autonomous regions) in China, with an average annual occurrence area of more than 106 hm^2^, and maize was the main host [9,10]. In addition, there is no diapause of *S. frugiperda*, and it cannot overwinter in the temperate areas in the north. As a result, it needs to make a round trip north and south every year in America [11,12,13]. As China and America have similar latitude and longitude, and similar climate, the Asian *S. frugiperda* population formed a seasonal migration pattern in East Asia, migrating from its year-round breeding area in the Indochina Peninsula and Southern China into Northern China, Japan and the Korean Peninsula [14,15,16], which is a severe threat to agricultural production and food security in China. In 2020, the Ministry of Agriculture and Rural Development of China listed *S. frugiperda* in *The list of First-class Crop Diseases and Pests* in China [17].

As a seasonal migratory pest, identifying the source distribution and migratory path of *S. frugiperda* is the basis for effective monitoring, early warning and control. Studies have shown that *S. frugiperda* in most regions of China and other temperate regions such as the Korean Peninsula and Japan cannot overwinter locally, and it needs to migrate northwards from Indochina peninsula and South China to the north of China, Japan and the Korean Peninsula for periodic damage every year [14,15,16,18]. Previous studies suggested that there are two migration pathways of *S. frugiperda*, eastern and western. The eastern pathway originates from Thailand, Laos, Vietnam and the year-round breeding area of China (Guangxi, Guangdong and Hainan provinces), and gradually migrates northward into the Yangtze River Valley, the Huang-Huai region (between the Huai River and the Yellow River) and the north of the Yellow River, and eventually migrated to Northeast China. The western pathway originates from Myanmar and the year-round breeding area of Yunnan Province, passes through Guizhou and Sichuan, and enters Shanxi, Shaanxi, Gansu and other provinces [14,19]. 

**Figure 1:**
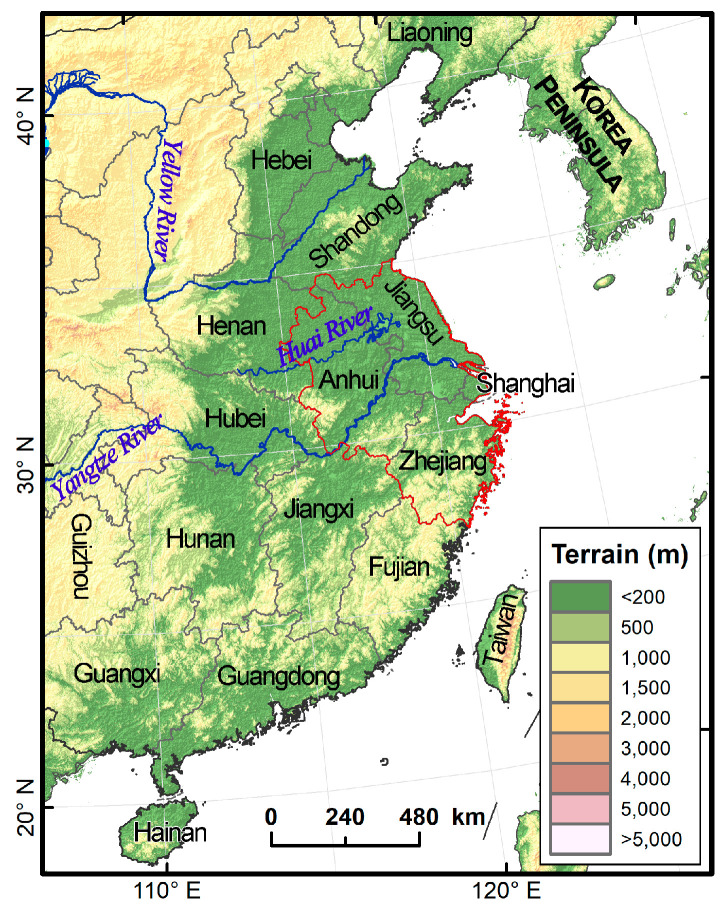
The migration arena of fall armyworm in East Asia. Most of East Asia is a large area of relatively flat land with few natural barriers to insect migration. Transported by the favorable winds associated with the movement of the Asian monsoon, invasive pest fall armyworm in this region takes regular, seasonal round-trip migration, as well as many other insects. The Yangtze River Delta located in East China, where the lower reaches of the Yangtze River meet the sea, is dominated by plain and includes Shanghai, Jiangsu, Zhejiang and Anhui provinces. Jiang–Huai region is the area between the Yangtze River and Huai river, rice is mostly transplanted after wheat is harvested in June. In the north of the Huai river, a large area of maize is planted in the summer, while a little maize is planted patchily in Jiang–Huai region and the south of the Yangtze River.

The Yangtze River Delta, located in East China, where the lower reaches of the Yangtze River meet the sea, is dominated by plain and includes Shanghai, Jiangsu, Zhejiang and Anhui provinces (Figure 1). The Yangtze River Delta plays an important strategic role in the overall pattern of agricultural development in China. It has a wide maize planting area, with an average annual maize planting area of 1.77 million hm^2^ from 2015 to 2019 (data from the National Bureau of Statistics of China, http://www.stats.gov.cn/tjsj/, accessed on 15 November 2022). At the same time, due to the flat terrain of the Yangtze River Delta and the impact of the typical East Asian monsoon climate, the Yangtze River Delta is an important passage for a variety of migratory pests, such as rice planthopper, *Mythimna separata* (Walker), *Cnaphalocrocis medinalis* Guenée and other major agricultural pests to migrate from south to north in the East Asian migratory fields [20,21,22], as well as a necessary passage and main landing area for *S. frugiperda* to migrate northward [23]. Chen et al. carried out a simulation by using the effective temperature accumulation law and found that the annual generation number of *S. frugiperda* in the Yangtze River Delta is 3~8 generations, and it could reach 6~8 generations in the south of the Yangtze River [19]. Similarly, according to the simulation results of the suitable areas for *S. frugiperda* in China, the Yangtze River Delta is the suitable area, and mainly the moderately suitable and highly suitable areas [24,25]. Therefore, there is a high risk of the outbreak of *S. frugiperda* in the Yangtze River Delta. In the first year of the invasion, more than 79.08% of the administrative districts and counties in the Yangtze River Delta were affected by *S. frugiperda* [9].

In addition, the Yangtze River Delta is a transitional area along the eastern migration pathway of *S. frugiperda*, connecting the year-round breeding area of South China and the Huang-Huai-Hai summer maize production area. The moths from Thailand, Vietnam and South China immigrate here in March or April, and begin to emerge in May and migrate out successively. In June, it reaches the peak of emigration, and the adults migrating northward can reach the Huang-Huai region and the north of the Yellow River [19]. In summary, once *S. frugiperda* breaks out in the Yangtze River Delta, it will not only cause serious losses to agricultural production such as maize, but also pose a severe threat to the Huang-Huai-Hai maize production area and the Northeast China spring maize production area. However, the current studies on the migration time and migration route of *S. frugiperda* in the Yangtze River Delta are only based on the simulation of its biological characteristics, and do not combine the actual field data for detailed analysis. Therefore, based on the high risk of *S. frugiperda* invasion and the important geographical location of the Yangtze River Delta, it is of great significance to clarify the migration dynamics of *S. frugiperda* in the Yangtze River Delta for the scientific prevention and control of *S. frugiperda* in the Yangtze River Delta, and even in North China and Northeast China.

In order to clarify the distribution of insect source, migration pathways and weather background field of *S. frugiperda* in the Yangtze River Delta, this study used the WRF-based insect three-dimensional trajectory analysis program to simulate the immigration and emigration trajectories of *S. frugiperda* in the Yangtze River Delta, and used R software to statistically analyze the probability of endpoints. Meanwhile, we used GrADS meteorological graphics software to display and explore the weather background field of *S. frugiperda*, and analyzed the influence of atmospheric background field on the migration process of *S. frugiperda*. Through the above research, we hope to provide a theoretical reference for the monitoring and early warning and scientific prevention and control of *S. frugiperda* in the Yangtze River Delta.

## 2. Materials and Methods

### 2.1. Data Sources

Insect survey data: The field survey data, light trapping data and sexual trapping data of *S. frugiperda* were all from the National Agro-Tech Extension Service Center of China. All these data recorded detail information of the first *S. frugiperda* observed in each site for each year, including the date and growth stage of *S. frugiperda* (Table S1). 

Meteorological data: The meteorological data used the Final Analysis (FNL) data from the National Centers for Environmental Prediction (NCEP) and the National Center for Atmospheric Research (NCAR) (https://rda.ucar.edu/datasets/ds083.2/index.html, accessed on 14 January 2022). These data is updated every 6 h and the spatial resolution is 1.0° × 1.0°.

### 2.2. Timing of Immigration and Emigration of S. frugiperda

In this study, we used the meteorological graphics processing software GrADS 2.1 (http://cola.gmu.edu/grads, accessed on 15 October 2022) to extract the temperature 2 m above the ground at 2:00 and 14:00 from the FNL data, which represent the daily minimum and maximum temperature respectively [7], so as to calculate the daily mean temperature. According to the daily mean temperature, discovery time and instar of *S. frugiperda* found in various regions of the Yangtze River Delta, the effective accumulated temperature required for egg stage and larva stage of *S. frugiperda* was used to calculate the immigration and emigration time. The effective accumulated temperatures from egg to adult were 35.72, 47.14, 26.86, 21.58, 24.78, 28.53, 32.57, 147.06 °D, respectively [26].

### 2.3. Migration Trajectory Analysis of S. frugiperda

#### 2.3.1. Meteorological Data Processing of WRF Model

The Weather Research and Forecasting (WRF, version 4.0, https://www2.mmm.ucar.edu/wrf/users/download/, accessed on 15 July 2022) model is a next-generation mesoscale numerical weather prediction system. In this study, FNL data was used as the meteorological data for the model inputs, and WRF model was used to generate the hourly meteorological element fields for three-dimensional trajectory analysis program to simulate the trajectory of *S. frugiperda*. Ma et al. [27] and Li et al. [14] were referred to for simulation scheme selection and parameters, as shown in Table 1.

#### 2.3.2. Trajectory Modelling of *S. frugiperda*

The three-dimensional trajectory analysis program based on the WRF model was used to calculate the migration trajectory of *S. frugiperda*. According to the migration behavior of *S. frugiperda*, the trajectory simulation parameters were set as follows: (1) Migration duration. Most noctuid moths migrate at night, and usually take off at dusk, land at the following dawn, and can fly for 3 nights [13,22,28]. Therefore, according to the sunrise and sunset time in the Yangtze River Delta, this paper set the takeoff time of *S. frugiperda* as 18:00 (BJT), and the landing time as 04:00 (BJT), with the flight duration of 10 h per night for 3 consecutive nights. When *S. frugiperda* flies over sea, such as the East China Sea and Yellow Sea, nocturnal flight duration is extended until it reaches land, but total flight does not exceed 36 h. (2) Flight speed. The self-flight speed of noctuids with similar body size is about 2.5–4.3 m/s [28,29], so the self-flight speed of *S. frugiperda* was set as 3 m/s. (3) Flight height. The initial height was set as 500 m, 750 m, 1000 m, 1250 m, 1500 m, 1750 m, 2000 m and 2250 m, with a total of 8 flight heights [13,23]. (4) The low temperature flight threshold. When migratory insects fly at high altitude, they will stop flying when the ambient temperature is lower than their low temperature flight threshold. The low temperature flight threshold of *S. frugiperda* was set as 13.8 °C [30], which has been widely used in previous studies [7,14,19].

#### 2.3.3. Trajectory Screening and Landing Probability Statistics of *S. frugiperda*

Spatial frequency distributions of valid trajectory endpoints were calculated and plotted by ArcGIS (Version 10.4, https://www.esri.com/en-us/home, accessed on 15 July 2022) and R (Version 4.0.3, https://www.r-project.org/, accessed on 15 July 2022). The main steps are as follows: Firstly, the endpoints on the sea and in the non-occurrence area of *S. frugiperda* were removed by ArcGIS to obtain the valid trajectory endpoints. Secondly, a cell grid of 1° × 1° was drawn which covered all valid endpoints, and used ArcGIS to calculate the number of valid trajectory endpoints in each cell. The number of cell points is arranged from small to large to calculate the cumulative probability. Finally, according to the cumulative probability, the spatial distribution of trajectory endpoints was drawn using R.

### 2.4. Meteorological Background Analysis

Based on FNL data, we used GrADS to plot the wind field at 850 hPa from April to July of the immigration period, the wind field at 850 hPa during June to August of the emigration period from 2019 to 2021, and the difference of monthly average temperature in Southern China from April to July in adjacent years. In addition, we also plotted the monthly cumulative precipitation map from April to July of 2019 to 2021 based on CRU TS v.4.06 data (https://crudata.uea.ac.uk/cru/data/hrg/cru_ts_4.06/, accessed on 27 September 2022).

## 3. Results

### 3.1. Immigrating Timing and Peak Period of S. frugiperda in the Yangtze River Delta

**Figure 2:**
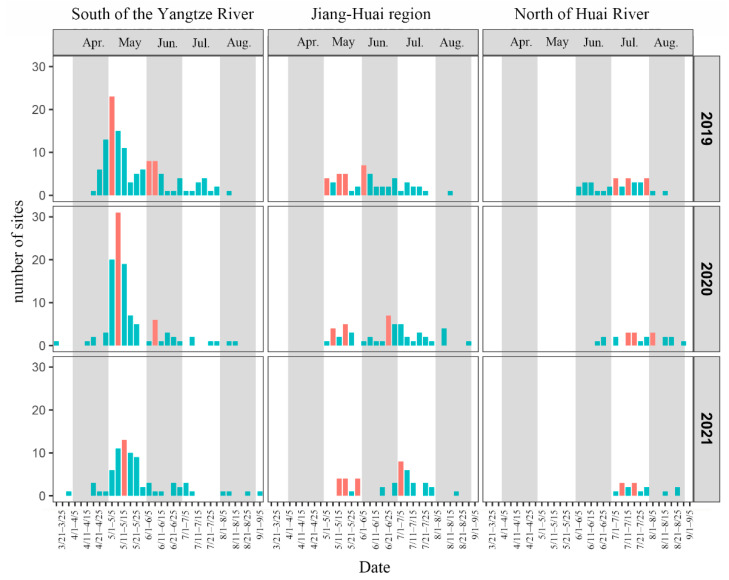
Immigrating timing distribution of *Spodoptera frugiperda* in the Yangtze River Delta from 2019 to 2021. The red bar in the figure represents the immigration peak.

By using the effective accumulated temperature model of *S. frugiperda*, the first immigrating time of *S. frugiperda* at each site in the south of the Yangtze River, the Jiang–Huai region (between the Huai River and the Yangtze River) and the north of Huai River from 2019 to 2021 was calculated, and summarized every five days (Figure 2). The results showed that *S. frugiperda* in the south of the Yangtze River began to immigrate in April. May is the main immigration period of *S. frugiperda* in the south of the Yangtze River, and the number of sites that *S. frugiperda* immigrated in May accounted for 51.22%, 75.23% and 68.00%, respectively, over the three years. At the same time, there was an obvious immigration peak in the south of the Yangtze River in May, but the time of immigration peak was delayed year by year. In June, there was also a small immigration peak in the south of the Yangtze River in 2019 and 2020, but no clear immigration peak in 2021. In addition, the first immigrating time of *S. frugiperda* in 2020 and 2021 was nearly one month earlier than that in 2019. Since May, *S. frugiperda* continued to move into the Jiang–Huai region, and the immigration peaks were scattered from May to July, and the time of immigration peak was delayed year by year. In 2021, the first immigration of *S. frugiperda* in the Jiang–Huai region was slightly delayed compared with the previous two years, and the first peak appeared the latest. In the north of Huai River, *S. frugiperda* gradually immigrated from June, and July was the main immigration period of *S. frugiperda*. The number of immigration sites in July accounted for 58.82%, 50.00% and 80.00% respectively, over the three years. The immigration peaks were mainly distributed in July. In addition, the time of first immigration to the north of Huai River was delayed year by year.

In summary, the main immigration period in the south of the Yangtze River was in May, and the immigration peaks were mainly in the early and middle of May. The immigration peaks of the Jiang–Huai region were scattered from May to July. The migration peaks in the north of Huai River were scattered, mainly in July. Compared with different years, the immigration peak in the same month of each region was delayed every year. According to the overall situation in the Yangtze River Delta, *S. frugiperda* moved to the south of the Yangtze River at the earliest, then gradually moved from south to north into the Jiang–Huai River region, and finally into the north of Huai River. 

### 3.2. Source Areas of S. frugiperda Migrating to the Yangtze River Delta

**Figure 3:**
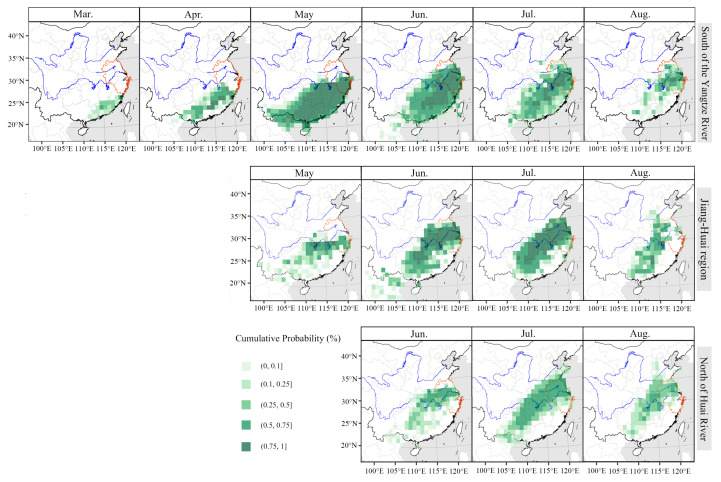
Backward trajectory endpoints distribution of *Spodoptera frugiperda* in the Yangtze River Delta from 2019 to 2021.

The backward trajectory endpoints of *S. frugiperda* in the Yangtze River Delta from 2019 to 2021 were calculated, as shown in Figure 3. In the south of the Yangtze River, *S. frugiperda* migrated for the first time in March or April, and its main source areas were Guangxi, Guangdong, Jiangxi and Fujian provinces. The main immigration period was May. In May, the valid endpoints of backward trajectory in the south of the Yangtze River were widely distributed, reaching as far south as Hainan Province and as far north as Hubei Province. The main insect sources were Guangxi, Guangdong, Fujian, Jiangxi and Hunan provinces. In June and July, the main source areas of *S. frugiperda* expanded northward and contracted southward. In addition to Zhejiang and Anhui in the Yangtze River Delta, the main source areas included Jiangxi and Hunan provinces. In August, the valid endpoints of backward trajectory were mainly distributed in Anhui, Zhejiang and Jiangxi provinces.

As for the Jiang–Huai region, in May, the endpoints were mainly distributed in Hunan, Jiangxi, Zhejiang and other places. Compared with the south of the Yangtze River in May, the distribution range of insect source areas shrank northward. In June and July, except for Jiangsu and Anhui in the Yangtze River Delta, the source areas were mainly distributed in northern Jiangxi, eastern Hubei and Hunan provinces, which were farther north than the source areas in the south of the Yangtze River. In August, the trajectory endpoints were scattered in eastern Guangxi, western Guangdong, Hunan, western Jiangxi, eastern Hubei and eastern Hebei provinces.

For the north of Huai River, the backward trajectory endpoints of *S. frugiperda* were centrally distributed in eastern Hubei, southern Anhui and southern Jiangsu provinces in June. In July, *S. frugiperda* migrated to the north of Huai River on a large scale, and its trajectory endpoints were concentrated in Jiangsu, Anhui, Hunan, Hubei, Henan and Guizhou provinces. In August, the backward trajectory endpoints of *S. frugiperda* were widely distributed in Hubei, Hunan, Henan, Jiangxi, Jiangsu and Anhui provinces.

Collectively, these results show that a small number of insect sources in the Yangtze River Delta moved into the south of the Yangtze River as early as March or April, and the main insect source areas were Guangxi, Guangdong, Jiangxi and Fujian provinces. In May of the main immigration period, the source areas of the south of the Yangtze River were mainly distributed in Guangxi, Guangdong, Fujian, Jiangxi and Hunan provinces. In May and June, *S. frugiperda* further migrated to the Jiang–Huai region, and the source areas were mainly distributed in Jiangxi, Hunan, Zhejiang, Jiangsu, Anhui and Hubei provinces. *S. frugiperda* migrated to the north of Huai River relatively late, and some of them moved in in June, but July was the main immigration period. Its insect source areas were mainly distributed in Jiangsu, Anhui, Hunan, Hubei, Henan and Guizhou provinces. From the south of the Yangtze River to the north of Huai River, the distribution of the main source areas of *S. frugiperda* continued to move northward.

### 3.3. Pupae Eclosion Time and Peak Period of of Immigrant Generation

**Figure 4:**
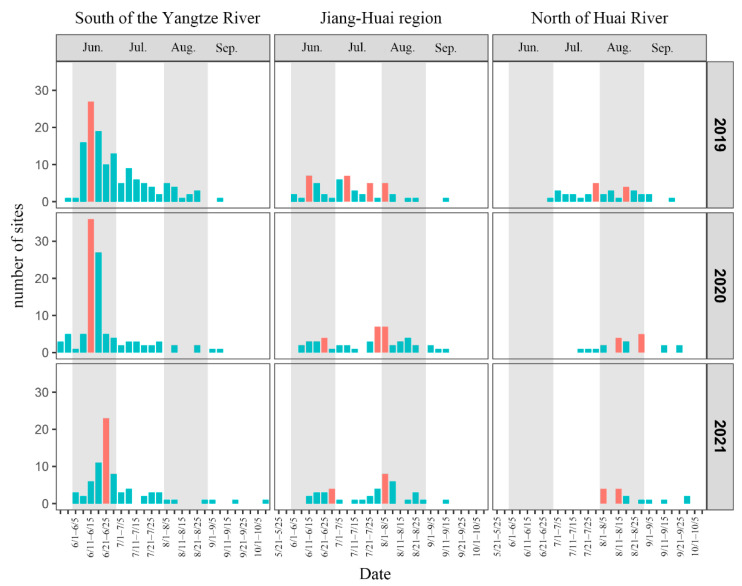
Eclosion time distribution of *Spodoptera frugiperda* in the Yangtze River Delta from 2019 to 2021. The red bar in the figure represents the eclosion peak.

Based on the effective accumulated temperature model of *S. frugiperda*, the pupae eclosion time of immigrant generation in the south of the Yangtze River, the Jiang–Huai region and the north of Huai River from 2019 to 2021 were calculated and classified every five days (Figure 4). In the south of the Yangtze River from 2019 to 2021, pupae eclosion began from the end of May at the earliest, and the eclosion time was concentrated in June, with 66.42%, 72.90% and 71.62% of the sites of *S. frugiperda* eclosion in June. The eclosion peaks were concentrated in mid to late June. In the Jiang–Huai region, *S. frugiperda* emerged from June, and the eclosion time in 2019 was concentrated in June (34.62%) and July (46.15%). In 2020 and 2021, the eclosion time was scattered, and the pupae eclosion continued from June to August, with eclosion sites accounting for 26.00%/30.00%/36.00% and 29.27%/21.95%/46.34%, respectively. There were eclosion peaks in June, July and August over the three years, and the time of eclosion peaks was delayed year by year. The eclosion time in the north of Huai River varied greatly from year to year. In 2019, pupae began to emerge at the end of June, and the eclosion time was mainly distributed in July and August, and the proportion of sites with eclosion time in July and August was just 44.12%. The eclosion time from 2020 to 2021 was concentrated in August, accounting for 66.67% and 73.33%, respectively. The eclosion peak over the three years mainly occurred in August.

Overall, the eclosion time of *S. frugiperda* in the south of the Yangtze River was the earliest, with the eclosion time concentrated in June and the peak time concentrated in middle and late June. The eclosion time in the Jiang–Huai region was later than that in the south of the Yangtze River. The concentrated eclosion time varied in different years, but was mainly in June, July and August. The peak period of eclosion was different in different years, which showed that the peak period was from mid-June to early August, but the distribution of the peak period was scattered. The eclosion time in the north of Huai River was the latest. Only in 2019, the eclosion time was concentrated in July and August, and the eclosion time in the other two years was concentrated in August. As for the peak period, only in late July 2019, the peak period of eclosion first appeared in the north of Huai River, and the peak period of eclosion in three years was mainly in August.

### 3.4. The Emigration Trajectory of Immigrant Generation of S. frugiperda

**Figure 5:**
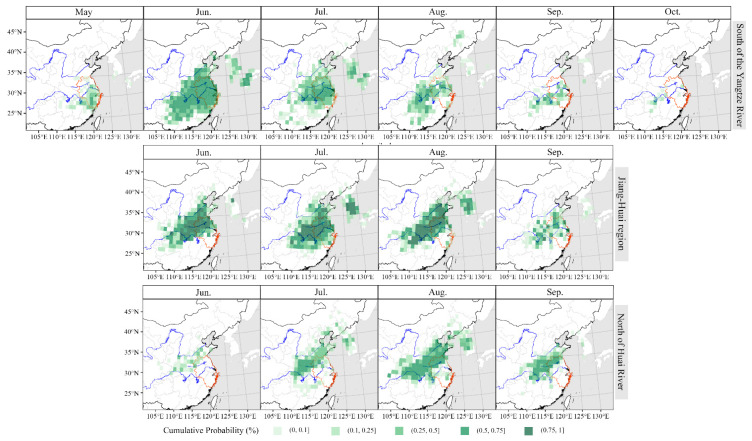
Forward trajectory endpoint distribution of *Spodoptera frugiperda* in the Yangtze River Delta from 2019 to 2021.

After the eclosion of *S. frugiperda*, supposing that some adults migrate out, the migration trajectory of *S. frugiperda* in various regions of the Yangtze River Delta was simulated to further analyze the distribution of its endpoints (Figure 5). The endpoints of the emigration trajectory of *S. frugiperda* in the south of Yangtze River were analyzed. In May, most of the endpoints were concentrated in the south of the Yangtze River, and a few in the Jiang–Huai region, northern Fujian and northern Jiangxi provinces. June was the large-scale emigration period. Besides Anhui and Jiangsu provinces in the Yangtze River Delta, *S. frugiperda* could also migrate to Hunan, Jiangxi, Hubei, Henan and Shandong provinces, etc., and could cross the sea to North Korea, South Korea and the coastal areas of Southwest Japan. In July, the trajectory endpoints were further north than in June, mainly concentrated in Anhui and Jiangsu in the Yangtze River Delta, some of which could reach Hubei, Henan and Shandong provinces, and could cross the sea to most areas of South Korea and the coastal areas of Southwest Japan. In August, emigration landing mainly occurred in Anhui, Jiangsu, Hunan and Hubei provinces, with a few likely to reach Jilin, Liaoning or even southern Heilongjiang province. In September and October, *S. frugiperda* in the south of the Yangtze River rarely migrated northward, and the endpoints were mainly distributed in the Jiang–Huai region of the Yangtze River Delta and Hunan and Hubei provinces.

In combination with the forward trajectory simulation of *S. frugiperda* in the Jiang–Huai region (Figure 4), in June, July and August, after the eclosion of *S. frugiperda*, except Anhui and Jiangsu provinces, *S. frugiperda* could migrate westward to Hubei and northern Hunan provinces, northward to Henan and Shandong provinces, as far as Hebei and Liaoning provinces. In addition, *S. frugiperda* could cross into North Korea, South Korea and Japan. In September, *S. frugiperda* mainly moved to Jiangsu, Anhui, Hubei, northern Hunan, southern Henan and southeastern Shandong provinces.

The forward trajectory of *S. frugiperda* in the north of Huai River (Figure 4) showed that some of the adults could migrate to Hubei, Henan and Shandong provinces as early as June. The main emigration period was in July and August. During this period, *S. frugiperda* could be moved to Hubei, Henan, Shandong and Hebei, and a few could reach Liaoning and Jilin and even cross the sea to North Korea and South Korea. In September, *S. frugiperda* decreased its northward migration, and mainly migrated to Hubei, Henan and Shandong provinces. From June to August, the range of northward migration of *S. frugiperda* gradually expanded, but narrowed in September.

In a word, apart from migrating to other areas in the Yangtze River Delta, *S. frugiperda* in the Yangtze River Delta can also provide insect sources to the surrounding provinces of Jiangxi, Hunan, Hubei, Henan, Shandong and Hebei. In July and August, *S. frugiperda* even crossed the Shandong Peninsula into Liaoning, Heilongjiang provinces in Northeast China. There were two main migration routes of *S. frugiperda* in the Yangtze River Delta, westward and northward. In addition, a small number of *S. frugiperda* migrated eastward across the sea to North Korea, South Korea and the coastal areas of southwest Japan from June to August.

### 3.5. Meteorological Background during the Migration of S. frugiperda

**Figure 6:**
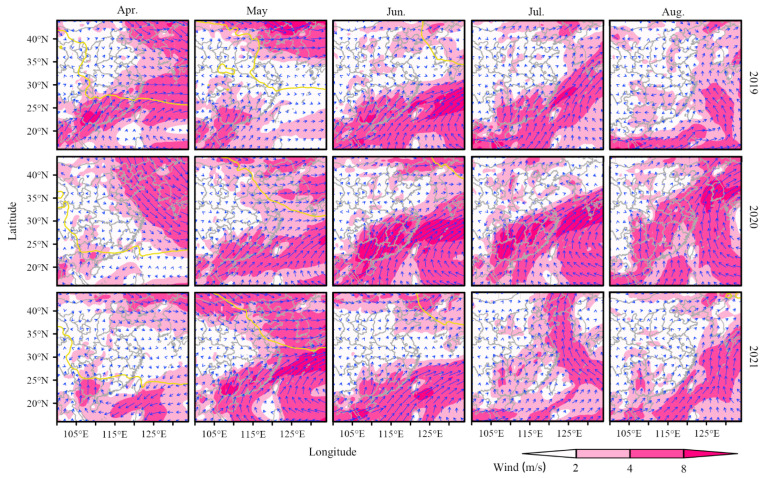
Average wind field at 850 hPa during the migration of *Spodoptera frugiperda* from April to August of 2019 to 2021. The yellow solid line is the 13.8 °C isotherm.

High-speed wind at high altitude is necessary for insects to complete their long-range migration. Therefore, we analyzed the wind field at 850 hPa in the south of China from 2019 to 2021 (Figure 6). All in all, the migration process of *S. frugiperda* was coincident with the development of the southwesterly airstream at 850 hPa. In April 2019, there was a strong southwest airflow at 850 hPa from Vietnam and Laos to the Yangtze River Delta through Guangdong and Guangxi. The wind speed was above 4 m/s, which was conducive to the northward migration of *S. frugiperda*. However, during the same time in 2020 and 2021, the southwesterly wind was weaker, and the north extension of the southwesterly wind was limited in South China (Figure 6), resulting in much fewer sites of *S. frugiperda* observed in April 2020 and 2021 than that in 2019 in the Yangtze River Delta (Figure 3). From May to July, the southwest airflow continued from Guangdong and Guangxi to the Yangtze River Basin at 850 hPa in Southern China. The wind speed remained at 2~8 m/s and even more over large area to the south of the Yangtze River, but wind is quick weak in Northern China, which encouraged the northward migration of *S. frugiperda* to the Yangtze River Delta, but not to further north (Figure 6). 

The first generation of *S. frugiperda* in the Yangtze River Delta emigrated mainly in June, while the wind direction over the Yangtze River Delta and its surrounding areas, such as Henan, Hebei and Shandong, was weak. As result, the emigrants of *S. frugiperda* did not take long distance migration, and mostly landed in surrounding areas (Figure 5). In July and August, southerly and southwesterly got stronger, which was convenient for *S. frugiperda* to migrate northward to Hebei, Shandong and other provinces, as well as cross the sea eastward to South Korea, North Korea and Japan at an appropriate time (Figure 5 and Figure 6). 

**Figure 7:**
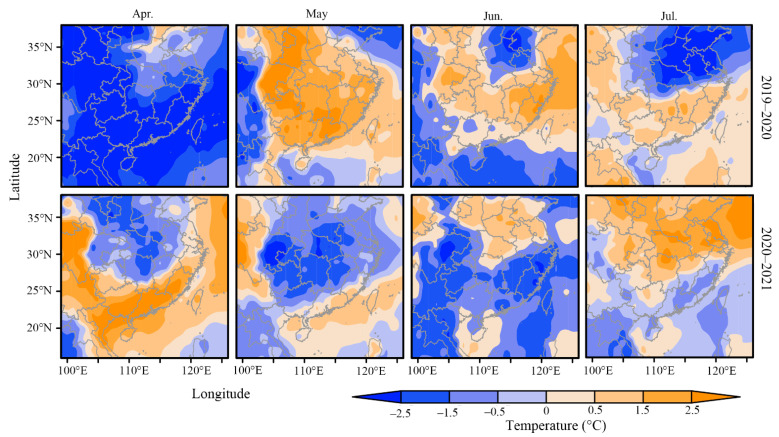
Difference of monthly average temperature in Southern China from April to July in adjacent year.

Temperature is an important environment factor to affect the migration process. Firstly, migrants cannot fly when the air temperature at flight altitude is below their flying low temperature threshold, such as 13.8 °C for *S. frugiperda*. The 13.8 °C isotherm in April 2020 and 2021 was more south than that in 2019 in April (Figure 6), that might stop the migration of *S. frugiperda* earlier, resulting in fewer moths moving to the Yangtze River Delta. Secondly, temperature is crucial for the growth and development of insects. In April, the monthly average temperature in Jiangxi, Hunan, Guangxi, Guangdong and Fujian provinces, where the source areas of the Yangtze River Delta in May, was more than 2.5 °C lower in 2020 than in 2019 (Figure 7). In May and June, the temperature of all in Southern China in 2021 was generally 0.5~2.5 °C lower than that in 2020, and locally more than 2.5 °C lower (Figure 7). The decrease in temperature would affect the growth and development of *S. frugiperda*, delay the eclosion time of pupae, and affect the emigration of *S. frugiperda* in the current month or even the next month, resulting in a later immigration peak from May to July in 2020 and 2021 (Figure 3). 

**Figure 8:**
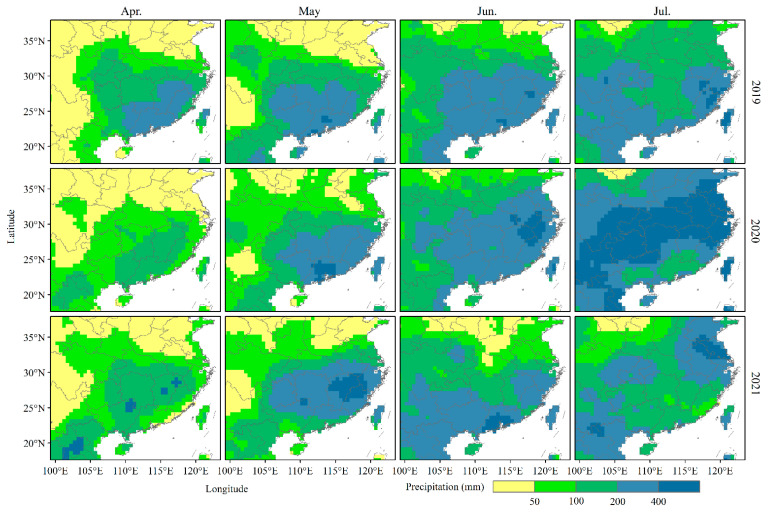
Monthly precipitation in Southern China from April to July of 2019 to 2021.

Rainfall always forms a barrier for terminating insect migrations, and thus the monthly precipitation of the past three years was analyzed (Figure 8). Most of Southern China was a rainy area in April–July. In May, the precipitation in Southern China increased, and the monthly precipitation in Guangdong in 2020 and Jiangxi and Fujian in 2021 even exceeded 400 mm. The rainy areas may affect the migration of *S. frugiperda* to the Yangtze River Delta to a certain extent, resulting in the delay of the immigration peak in May 2020 and 2021. In June, Jiangxi and northern Fujian in 2020 and Guangdong in 2021 also had high precipitation, and the barrier of rainy areas may affect the migration of *S. frugiperda* to the Yangtze River Delta. In July, the precipitation of cities along the Yangtze River in 2020 was above 400 mm, which may not only affect the immigration of *S. frugiperda*, but also affect the emigration.

## 4. Discussion

The Yangtze River Delta, as the migration transition zone of the eastern pathway of *S. frugiperda*, is connected with their year-round breeding area in South China and the main maize-producing area in Northern China. Therefore, this area is an important position for the prevention and control of *S. frugiperda* in China. In this study, compared with the first year of 2019, the first immigration time of *S. frugiperda* in 2020 and 2021 was earlier. This may be related to the fact that there are year-round breeding areas in Guangdong, Guangxi and Fujian provinces in China [15], which can provide insect sources in advance. *S. frugiperda* began to immigrate to the Yangtze River Delta in March or April, and the main immigration period was from May to July. Additionally, from south to north, it successively moved to the south of the Yangtze River, the Jiang–Huai region and the north of the Huai River. These results are in agreement with the findings of Chen et al. [19]. 

The results of this study showed that *S. frugiperda* in the Yangtze River Delta mainly migrated from the year-round breeding areas such as Guangxi, Guangdong, Fujian provinces, and the migration transition areas such as Jiangxi, Hunan, Hubei and Henan provinces. This is part of the eastern pathway of *S. frugiperda* in China, which coincides with the eastern pathway from Southeast Asia and China’s year-round breeding areas to the Huang-Huai-Hai Plain via the south of Yangtze River [14,19]. At the same time, a small amount of the backward trajectory endpoints in the Yangtze River Delta also fell in Yunnan and Guizhou on the western pathway. Through the analysis of the source areas, we found that *S. frugiperda* in the Yangtze River Delta mainly came from the source areas of South China, East China and Central China, and rarely from Vietnam, Laos and Thailand. Therefore, the effective prevention and control of *S. frugiperda* in the year-round breeding area of South China can greatly reduce the risk of its migration to the Yangtze River Delta and the Huang-Huai-Hai Plain.

Several reports have shown that *S. frugiperda* in China is mainly of the corn strain [31,32,33], mainly feeds on corn with a large appetite and strong reproductive ability. Moreover, as a typical migratory pest, *S. frugiperda* usually breaks out when it lands. As the transition zone of *S. frugiperda* in the eastern pathway, the Yangtze River Delta is connected with the year-round breeding area of *S. frugiperda* in Southern China and the two maize-producing areas of the Huang-Huai-Hai maize-producing area and the spring maize-producing area in Northeast China. In the Huang-Huai-Hai region, the spring maize can be sown near the grain rain and harvested at the end of August, summer maize is sown after wheat harvest and harvested in October. The spring maize-producing area in Northeast China can be sown in late April and early May and harvested in late August. Based on the emigration of *S. frugiperda* in the Yangtze River Delta from 2019 to 2021, the northward migration trend of *S. frugiperda* slowed down after August, and the time of emigration to the Northeast China was also concentrated in August. At the same time, considering the decrease in temperature and lack of host plants in Northern China after August, *S. frugiperda* posed a small threat to the spring maize-producing area in Northeast China. The difference is that the risk of *S. frugiperda* infestation is high in the Huang-Huai-Hai maize-producing area. The reason is that the Huang-Huai-Hai region is the end point of northward migration and the starting point of southward migration. In addition, the spring maize and summer maize in this region are mixed from June to August, so the maize at different growth stages can continuously provide food for *S. frugiperda*.

In reviewing the literature, Chen et al. analyzed the source areas of *S. frugiperda* first discovered in Shandong in 2019, and suggested that the source areas were Jiangsu and Anhui provinces in the Yangtze River Delta [34]. Similarly, Sun et al. analyzed the backward trajectory of *S. frugiperda* in Henan in 2019 and 2020, and found that Jiangsu and Anhui in the Yangtze River Delta could also provide insect sources for Henan [35]. Consistent with the above literature, this research found that *S. frugiperda* in the Yangtze River Delta can be the source for *S. frugiperda* migrating to Shandong and Henan provinces. Moreover, the trajectory simulation of this research showed that from June to August, *S. frugiperda* in the Yangtze River Delta could cross the sea to the Korean Peninsula and the coastal areas in Southwest Japan. Relatedly, Ma et al. analyzed the risk of *S. frugiperda* invading South Korea and Japan in 2019, and concluded that *S. frugiperda* population is very easy to invade during the rainy season from June 1 to July 15 [27], which partially coincides with the migration time from June to August in this paper. Subsequently, Wu et al. simulated the backward trajectory of *S. frugiperda* in Korea and Japan, showing that from late May to late June, *S. frugiperda* in Zhejiang, Anhui, Jiangxi, Guangdong and Taiwan provinces can migrate cross the sea to Korea and Japan [36]. The result of this literature can support the result mentioned earlier in this paper that the Yangtze River Delta provided insect sources for Korea and Japan.

Most insects are too small to migrate a long distance only by their own flight capability, which is mainly by wind [28,37,38,39]. The results of Qi et al. and Chen et al. showed that the long-distance migration of *S. frugiperda* was related to the low-level jet [7,40]. Consistent with the literature, this research found that when there was a large-scale immigration of *S. frugiperda* in the Yangtze River Delta from May to July, there was a southwest low-level jet running through South China to the Yangtze River Delta at 850 hPa, which provided an effective carrier airflow for the migration of *S. frugiperda*. Different from the wind, precipitation often hinders the migration of moths [41].

The Yangtze River Delta is located in an important migration site in East Asia. It is necessary to prevent the grassland armyworm from invading the area south of the Yangtze River from late March, and to monitor, warn and effectively control the grassland armyworm in the annual breeding area of South China, so as to prevent the migration of the grassland armyworm in the annual breeding area of South China. In the main migration period from May to July, we can closely combine the situation of the grassland armyworm in the Yangtze River Delta and its main source areas to carry out monitoring and early warning of the grassland armyworm and timely prevention and control. Once the grassland noctuid infestation occurs in the Yangtze River Delta, we should be alert to its further invasion in Hubei, Shandong, Henan and Hebei from June to August.

The Yangtze River Delta is located in the key area of the East Asian migration field. It is necessary to prevent the invasion of *S. frugiperda* into the south of the Yangtze River from late March, and carry out the monitoring, early warning and effective prevention and control of *S. frugiperda* in the year-round breeding area of South China to prevent its emigration. In the main migration period from May to July, we can closely combine the situation of *S. frugiperda* in the Yangtze River Delta and its main source areas to carry out monitoring and early warning and timely prevention and control. Once *S. frugiperda* appears in the Yangtze River Delta, we should be alert to its further invasion to Hubei, Shandong, Henan and Hebei provinces from June to August.

## Figures and Tables

**Table 1:** Selection of scheme and parameters of the WRF model.

Item	Domain 1
Location	30 °N, 60 °E
The number of grid points	125 × 115
Distance between grid points (km)	60
Layers	32
Map projection	Lambert
Microphysics scheme	WSM6
Longwave radiation scheme	RRTMG
Shortwave radiation scheme	RRTMG
Surface layer scheme	Monin–Obukhov
Land/water surface scheme	Noah
Planetary boundary layer scheme	YSU
Cumulus parameterization	Tiedtke
Forecast time	72 h

## Data Availability

All the data generated from the current work are available upon reasonable request to the corresponding authors.

## Supplementary Materials

The following supporting information can be downloaded at: https://www.mdpi.com/article/10.3390/insects14020127/s1, Table S1: The detail information of the first *S. frugiperda* observed in the Yangtze River Delta from 2019 to 2021.
